# Supplementary material for: Genome-Wide Identification of the Aconitase Gene Family in Tomato (Solanum lycopersicum) and CRISPR-Based Functional Characterization of SlACO2 on Male-Sterility
Source: Int J Mol Sci. 2022 Nov 12;23(22):13963. doi: 10.3390/ijms232213963 (PMC9699144; doi:10.3390/ijms232213963)
Supplement: Supplementary file 1 [file ijms-23-13963-s001.zip › ijms-2035388-supplementary.pdf]

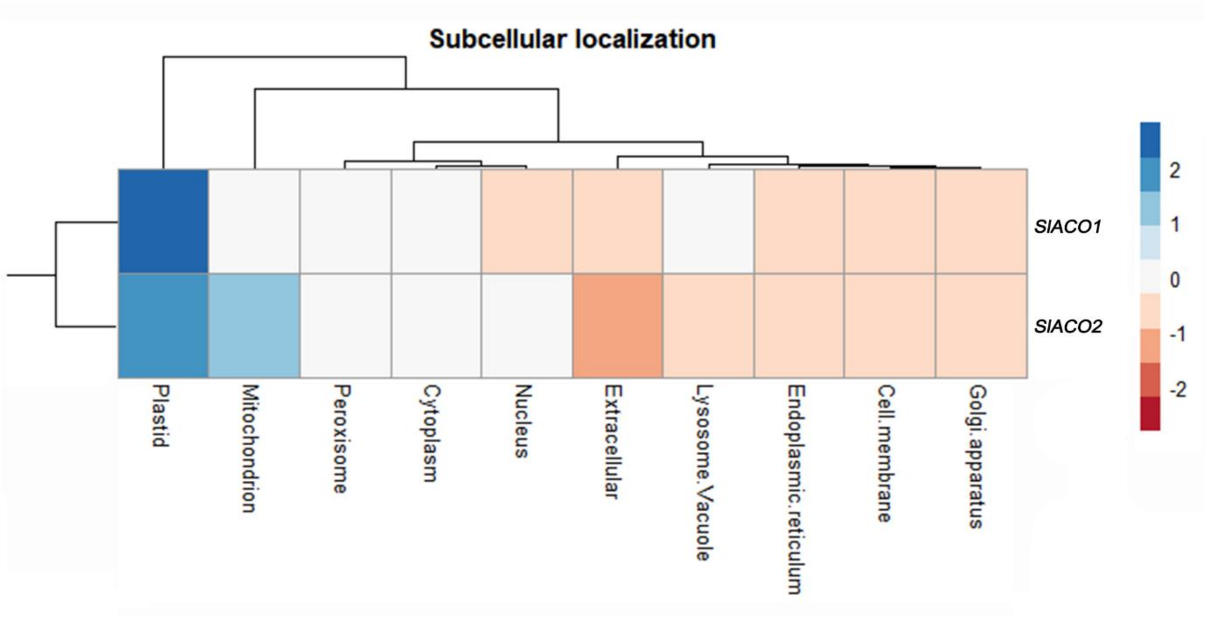

Supplementary Figure S1. Sub-cellular location of SlACO proteins.

a

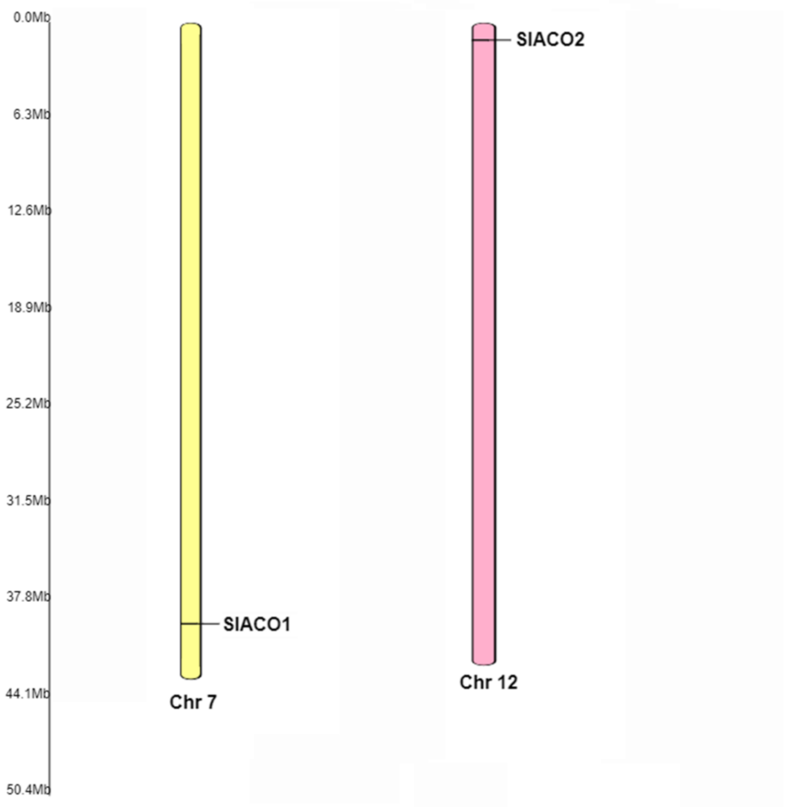

b

LOCUS_1	LOCUS_2	Ka	Ks	Ka/KS	Duplication	Time (MYA)
SlACO1	SlACO2	0.0628	0.5448	0.115272	Segmental	0.665

Supplementary Figure *S2*: Chromosomal location and duplication pattern of *SlACOs*.

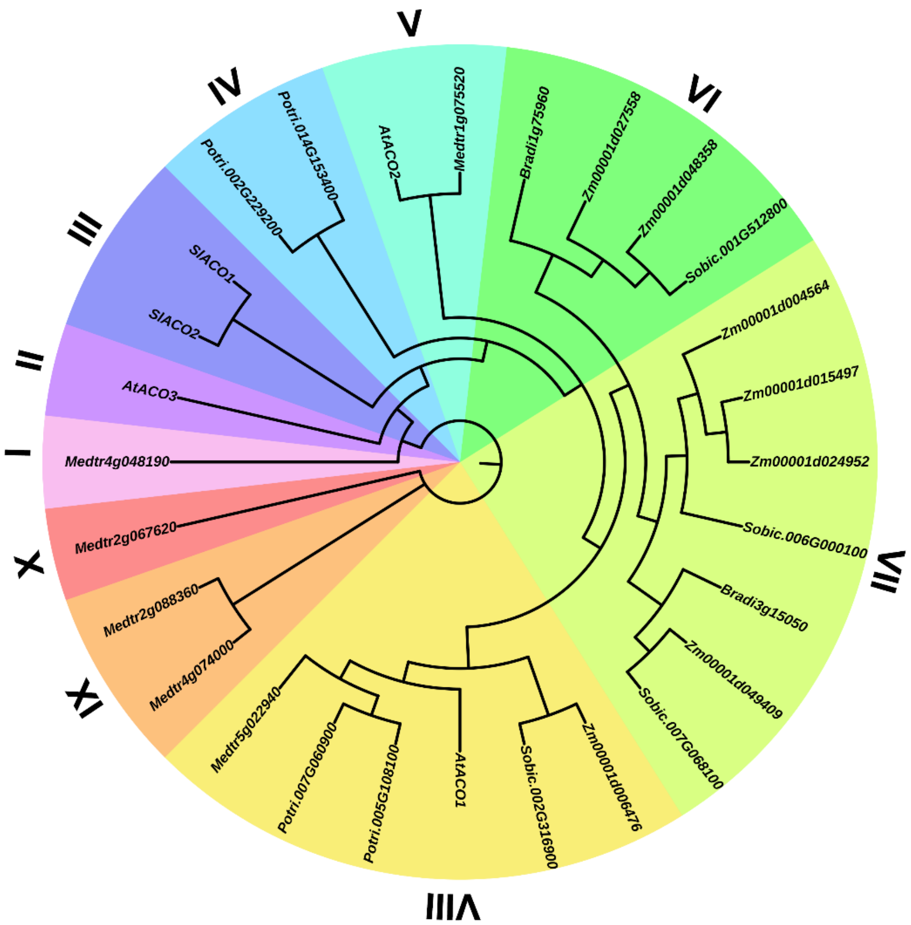

Supplementary Figure *S*3: Phylogenetic analysis of *ACO* genes in different organisms.

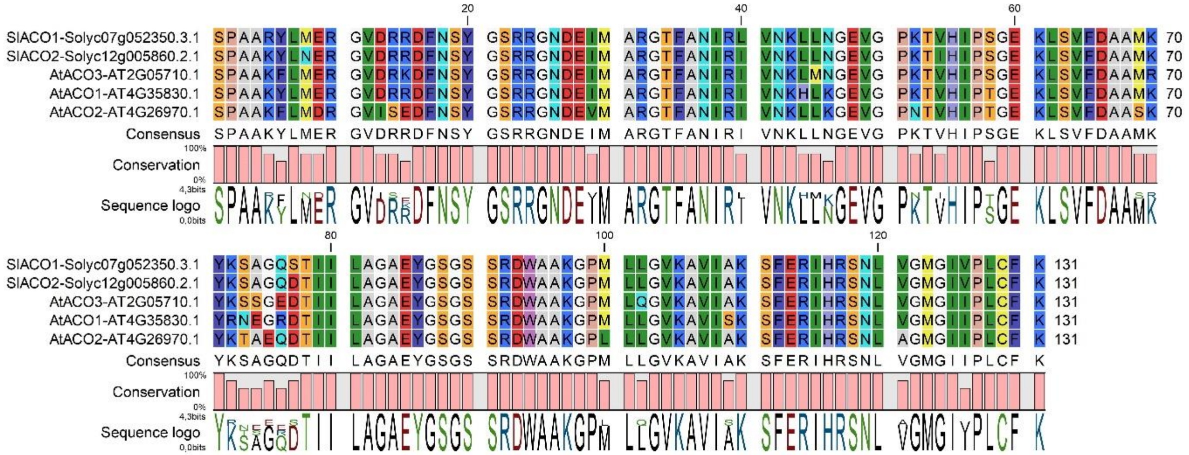

Supplementary Figure S4: Alignment of aconitase swivel domains of AtACOs and SlACOs proteins.

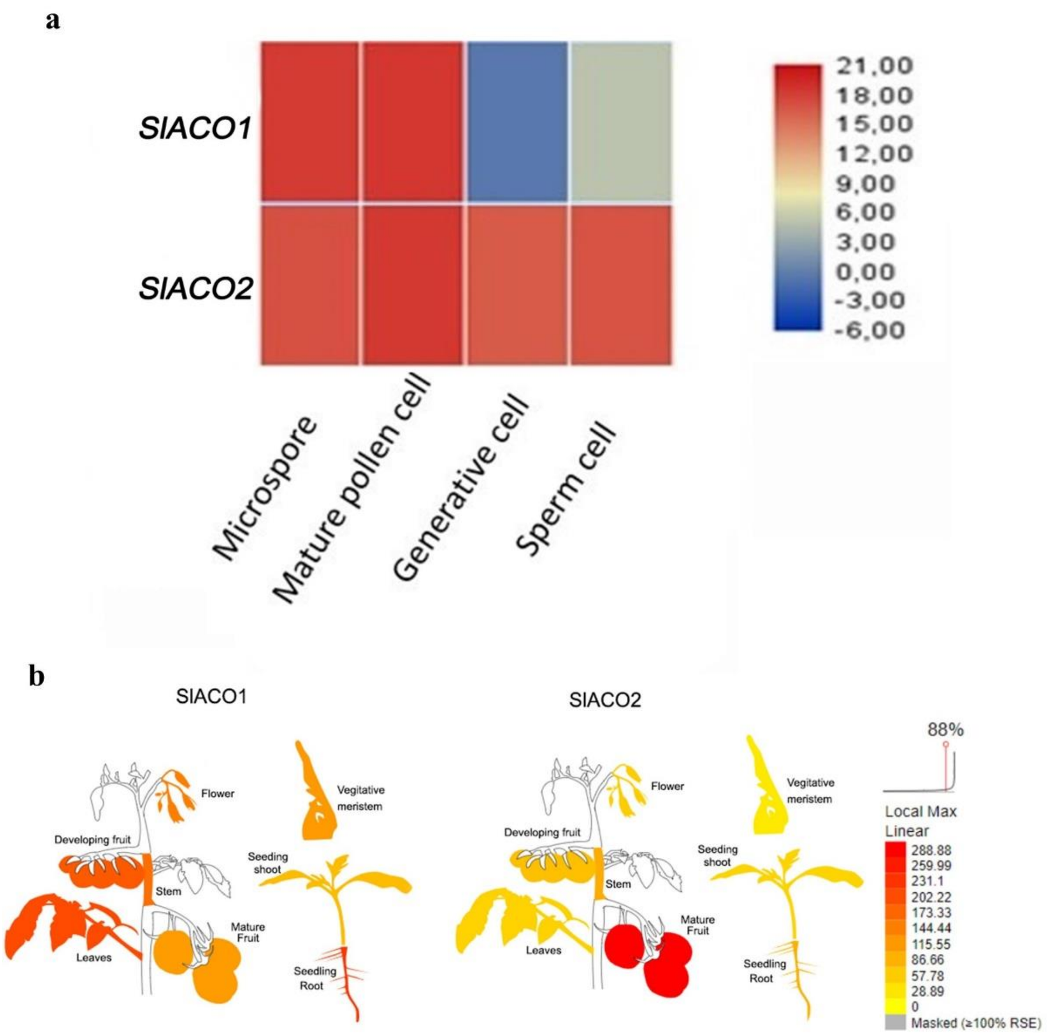

Supplementary Figure S5: Expression profiles of *SlACOs* in various tissues and organs. a) Expression analysis of *SlACOs* using RNA-seq data b) Organ and tissue-specific expression of *SlACOs*

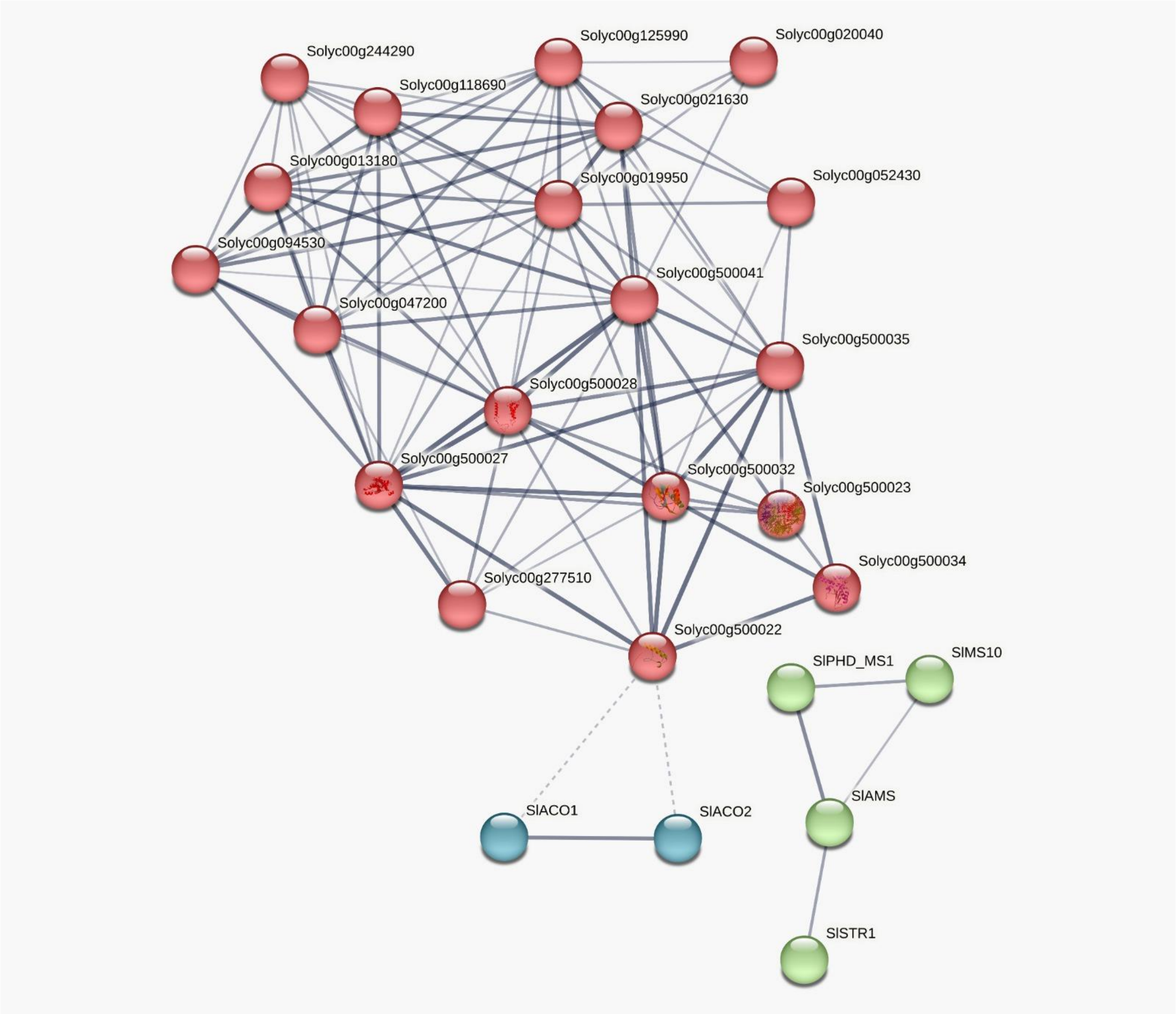

Supplementary Figure S6: Protein-protein interaction network of SlACOs.

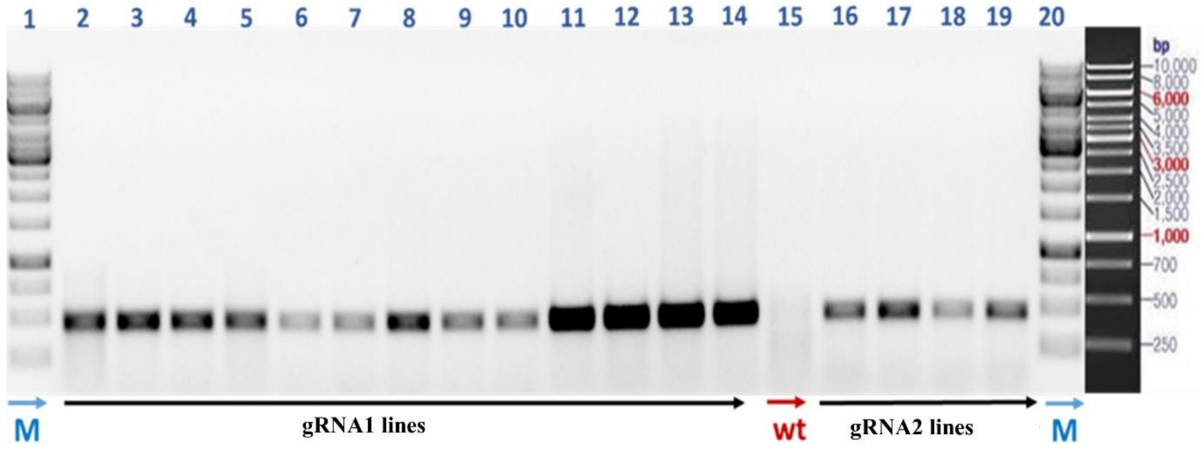

Supplementary Figure S7: PCR confirmation of SlACO lines using *hptII*-specific primers.

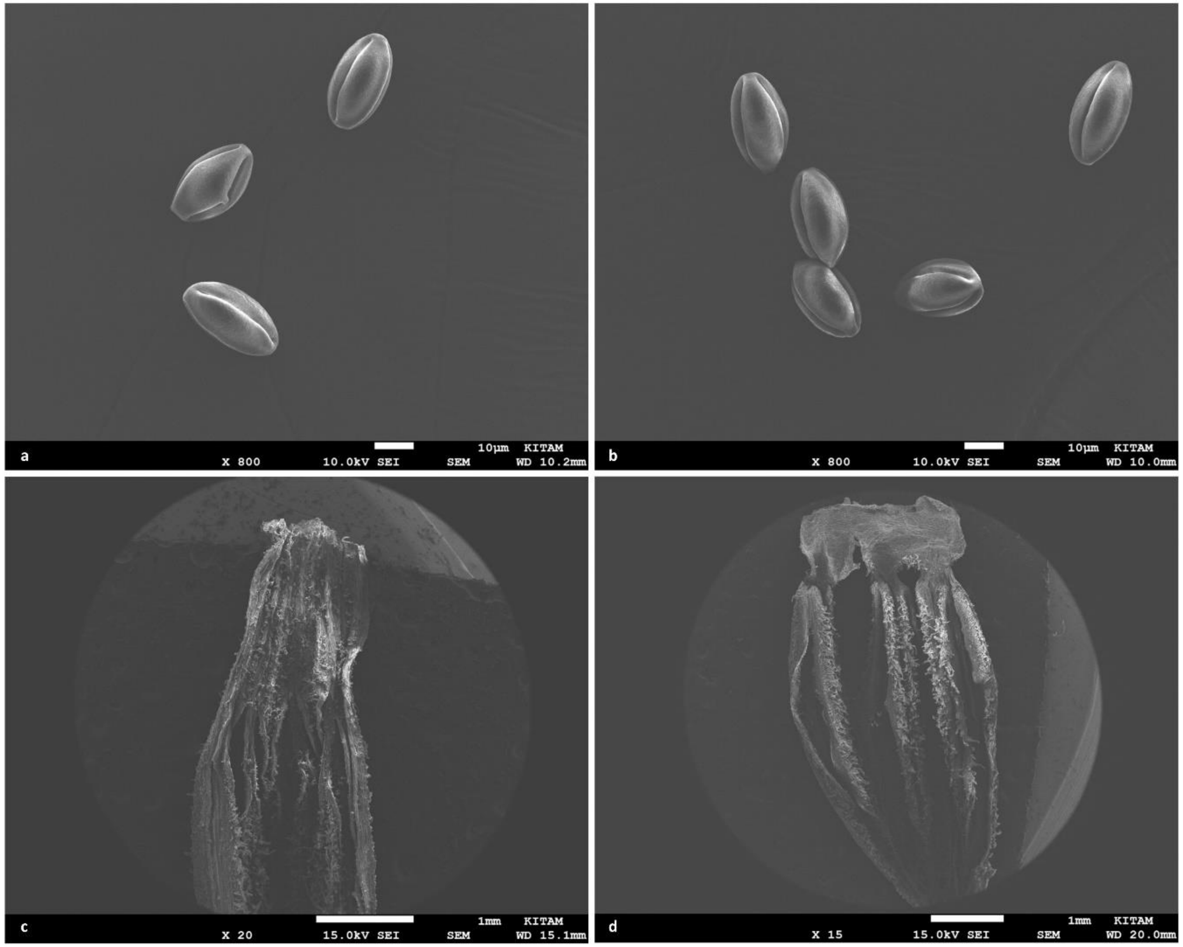

Supplementary Figure S8: Scanning electron microscope images of a)pollen grains and b) anthers.

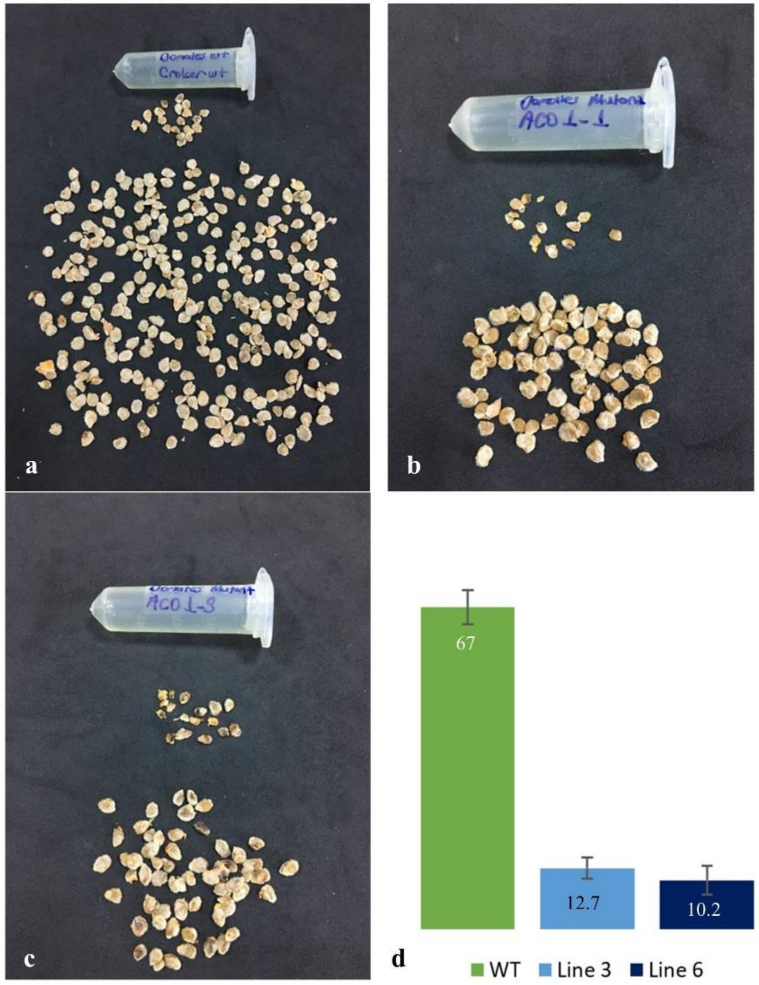

Supplementary Figure S9: Seed statics of WT and mutant plants. a) Seeds obtained from WT plants b)Line 3 c)Line 6 d) Graphic showing the seed numbers.

Supplementary Table S1: MCL Clusters of SlACOs with genes related to pollen development and male-sterility.

#clustering method	cluster number	cluster color	gene count	protein name	protein identifier	protein description
MCL	1	Red	17	Solyc00g013180	Solyc00g013180.1.1	annotation not available
MCL	1	Red	17	Solyc00g019950	Solyc00g019950.1.1	Nadh dehydrogenase (ubiquinone) fe-s protein 3; Belongs to the complex I 30 kDa subunit family
MCL	1	Red	17	Solyc00g020040	Solyc00g020040.1.1	Uncharacterized protein loc101259037; ABC-type heme transporter subunit
MCL	1	Red	17	Solyc00g021630	Solyc00g021630.1.1	Nadh-ubiquinone oxidoreductase chain 6; Core subunit of the mitochondrial membrane respiratory chain NADH dehydrogenase (Complex I) t
MCL	1	Red	17	Solyc00g047200	Solyc11g056490.1.1	annotation not available
MCL	1	Red	17	Solyc00g052430	Solyc00g052430.2.1	annotation not available
MCL	1	Red	17	Solyc00g094530	Solyc00g094530.1.1	annotation not available
MCL	1	Red	17	Solyc00g118690	Solyc00g118690.1.1	annotation not available
MCL	1	Red	17	Solyc00g125990	Solyc00g125990.1.1	annotation not available
MCL	1	Red	17	Solyc00g244290	Solyc00g244290.1.1	annotation not available
MCL	1	Red	17	Solyc00g277510	Solyc00g277510.1.1	annotation not available
MCL	1	Red	17	Solyc00g500027	Solyc01g007530.2.1	Cytochrome b6; Component of the cytochrome b6-f complex, which mediates electron transfer between photosystem II (PSII) and photosystem I
MCL	1	Red	17	Solyc00g500028	Solyc01g007540.2.1	Cytochrome b6/f complex subunit iv; Cytochrome b6-f complex subunit 4; Component of the cytochrome b6-f complex, which mediates electror
MCL	1	Red	17	Solyc00g500041	Solyc11g021230.1.1	NAD(P)H-quinone oxidoreductase subunit 1, chloroplastic; NDH shuttles electrons from NAD(P)H:plastoquinone, via FMN and iron-sulfur (Fe conserves the redox energy in a proton gradient; Belongs to the complex I subunit 1 family
MCL	1	Red	17	Solyc12g005630.1.1	Solyc12g005630.1.1	Cytochrome b6-f complex iron-sulfur subunit, chloroplastic; Cytochrome b6-f complex iron-sulfur subunit; Photosynthetic electron transfer C
MCL	1	Red	17	petA	Solyc01g007380.1.1	Apocytochrome f; Cytochrome f; Component of the cytochrome b6-f complex, which mediates electron transfer between photosystem II (PSII) a
MCL	1	Red	17	petG	Solyc01g007430.2.1	Cytochrome b6-f complex subunit 5; Component of the cytochrome b6-f complex, which mediates electron transfer between photosystem II (PSI
MCL	2	Yellow	9	Solyc00g021640	Solyc00g021640.2.1	annotation not available
MCL	2	Yellow	9	Solyc00g500022	Solyc01g007460.2.1	30S ribosomal protein S18, chloroplastic ; Belongs to the bacterial ribosomal protein bS18 family
MCL	2	Yellow	9	Solyc00g500023	Solyc01g007490.2.1	ATP-dependent Clp protease proteolytic subunit; Cleaves peptides in various proteins in a process that requires ATP hydrolysis. Has a chymotr
MCL	2	Yellow	9	Solyc00g500032	Solyc01g007600.2.1	50S ribosomal protein L16, chloroplastic ; Belongs to the universal ribosomal protein uL16 family
MCL	2	Yellow	9	Solyc00g500034	Solyc01g007610.2.1	30S ribosomal protein S3, chloroplastic ; Belongs to the universal ribosomal protein uS3 family
MCL	2	Yellow	9	Solyc00g500035	Solyc01g007630.2.1	50S ribosomal protein L2, chloroplastic ; Belongs to the universal ribosomal protein uL2 family
MCL	2	Yellow	9	Solyc04g049260.1.1	Solyc04g049260.1.1	annotation not available
MCL	2	Yellow	9	Solyc08g006840.2.1	Solyc08g006840.2.1	annotation not available
MCL	2	Yellow	9	rps16	Solyc07g008660.2.1	30S ribosomal protein S16, chloroplastic; 30S ribosomal protein S16
MCL	3	Green	8	SlACO1	Solyc07g052350.2.1	Aconitate hydratase, cytoplasmic; Aconitate hydratase; Catalyzes the isomerization of citrate to isocitrate via cis-aconitate

MCL	3	Green	8	SlACO2	Solyc12g005860.1.1	Aconitate hydratase, cytoplasmic; Aconitate hydratase; Catalyzes the isomerization of citrate to isocitrate via cis-aconitate
MCL	3	Green	8	Solyc01g073740.2.1	Solyc01g073740.2.1	Citrate synthase, mitochondrial; Belongs to the citrate synthase family
MCL	3	Green	8	Solyc05g009030.2.1	Solyc05g009030.2.1	3-isopropylmalate dehydrogenase, chloroplastic; 3-isopropylmalate dehydrogenase; Catalyzes the oxidation of 3-carboxy-2-hydroxy-4- methylp
MCL	3	Green	8	Solyc07g055840.2.1	Solyc07g055840.2.1	annotation not available
MCL	3	Green	8	Solyc12g011000.1.1	Solyc12g011000.1.1	annotation not available
MCL	4	Blue	4	SlAMS	Solyc08g062780.1.1	Transcription factor aborted microspores isoform x2; Uncharacterized protein; Basic helix-loop-helix (bHLH) DNA-binding superfamily protein
MCL	4	Blue	4	SlMS10	Solyc02g079810.1.1	Transcription factor dyt1; Uncharacterized protein; Basic helix-loop-helix (bHLH) DNA-binding superfamily protein
MCL	4	Blue	4	SlPHD_MS1	Solyc04g008420.1.1	Phd finger protein male sterility 1; RING/FYVE/PHD zinc finger superfamily protein
MCL	4	Blue	4	SlSTR1	Solyc03g053130.2.1	Protein strictosidine synthase-like 13; Uncharacterized protein; Calcium-dependent phosphotriesterase superfamily protein

Supplementary Table S2: Predicted functional partners of SlACOs.		
**Gene ID or Name**	**Description**	**Score**
petA	Apocytochrome f; Cytochrome f; Component of the cytochrome b6-f complex, which mediates electron transfer between photosystem II (PSII) and photosystem I (PSI), cyclic electron flow around PSI, and state transitions	0.999
petG	Cytochrome b6-f complex subunit 5; Component of the cytochrome b6-f complex, which mediates electron transfer between photosystem II (PSII) and photosystem I (PSI), cyclic electron flow around PSI, and state transitions. PetG is required for either the stability or assembly of the cytochrome b6-f complex	0.999
Solyc01g073740.2.1	Citrate synthase, mitochondrial; Belongs to the citrate synthase family	0.999
Solyc05g009030.2.1	3-isopropylmalate dehydrogenase, chloroplastic; 3-isopropylmalate dehydrogenase; Catalyzes the oxidation of 3-carboxy-2-hydroxy-4- methylpentanoate (3-isopropylmalate) to 3-carboxy-4-methyl-2- oxopentanoate. The product decarboxylates to 4-methyl-2 oxopentanoate	0.999
Solyc07g055840.2.1	annotation not available	0.999
Solyc12g005630.1.1	Cytochrome b6-f complex iron-sulfur subunit, chloroplastic; Cytochrome b6-f complex iron-sulfur subunit; Photosynthetic electron transfer C	0.999
Solyc12g011000.1.1	annotation not available	0.999
Solyc04g049260.1.1	annotation not available	0.998
rps16	30S ribosomal protein S16, chloroplastic; 30S ribosomal protein S16	0.998
Solyc08g006840.2.1	annotation not available	0.998

Supplementary Table S3: Changes in pollen viability in WT and SlACO2.			
Lines	Pollen activity		
	active %	semi-active %	sterile %
WT	85.10	10.63	4.25
Line 3	4.00	12.00	84.00
Line 6	2.00	11.00	87.00
